# Supplementary material for: Static charge is an ionic molecular fragment
Source: Nat Commun. 2024 Mar 5;15:1986. doi: 10.1038/s41467-024-46200-3 (PMC10914821; doi:10.1038/s41467-024-46200-3)
Supplement: Supplementary file 1 — Supplementary Information [file 41467_2024_46200_MOESM1_ESM.pdf]

Supplementary Information

Static Charge is an Ionic Molecular Fragment

Yan Fang,[1,2†] Chi Kit Ao,[1†] Yan Jiang,[1] Yajuan Sun,[1] Linfeng Chen,[1] and Siowling Soh[1]*

[1] Department of Chemical and Biomolecular Engineering, National University of Singapore, 4 Engineering Drive 4, Singapore 117585, Singapore

[2] College of Biotechnology and Pharmaceutical Engineering, State Key Laboratory of Materials-Oriented Chemical Engineering, Nanjing Tech University, 30# Puzhu South Road, Nanjing, 211816, P.R. China

[†] These authors contributed equally to this work

* To whom correspondence may be addressed: chessl@nus.edu.sg

Supplementary Table 1. XPS elemental analyses of the uncoated surface ("Uncoated") and alkylsilane-coated surface with *n* =8 ("Coated with *n* = 8"). The large increase in the percentage of carbon on the alkylsilane-coated surface compared to the uncoated surface indicated that the alkylsilanes were successfully coated onto the surface of mica.

	Samples	O/%	C/%	K/%	Al/%	Si/%
Before Contact	Uncoated	59.4	9.3	3.7	14.5	13.2
	Coated with *n* = 8	40.0	33.8	2.7	14.0	9.5

Supplementary Table 2. Root-mean-square (RMS) surface roughness of the mica coated with the alkylsilanes of a specific number of carbon atoms, *n* (i.e., before using them for contact electrification). *n* = 0 represents the case when the mica was not coated.

n	RMS (nm)
0	0.19
1	0.59
2	0.44
4	0.49
6	0.46
8	0.54

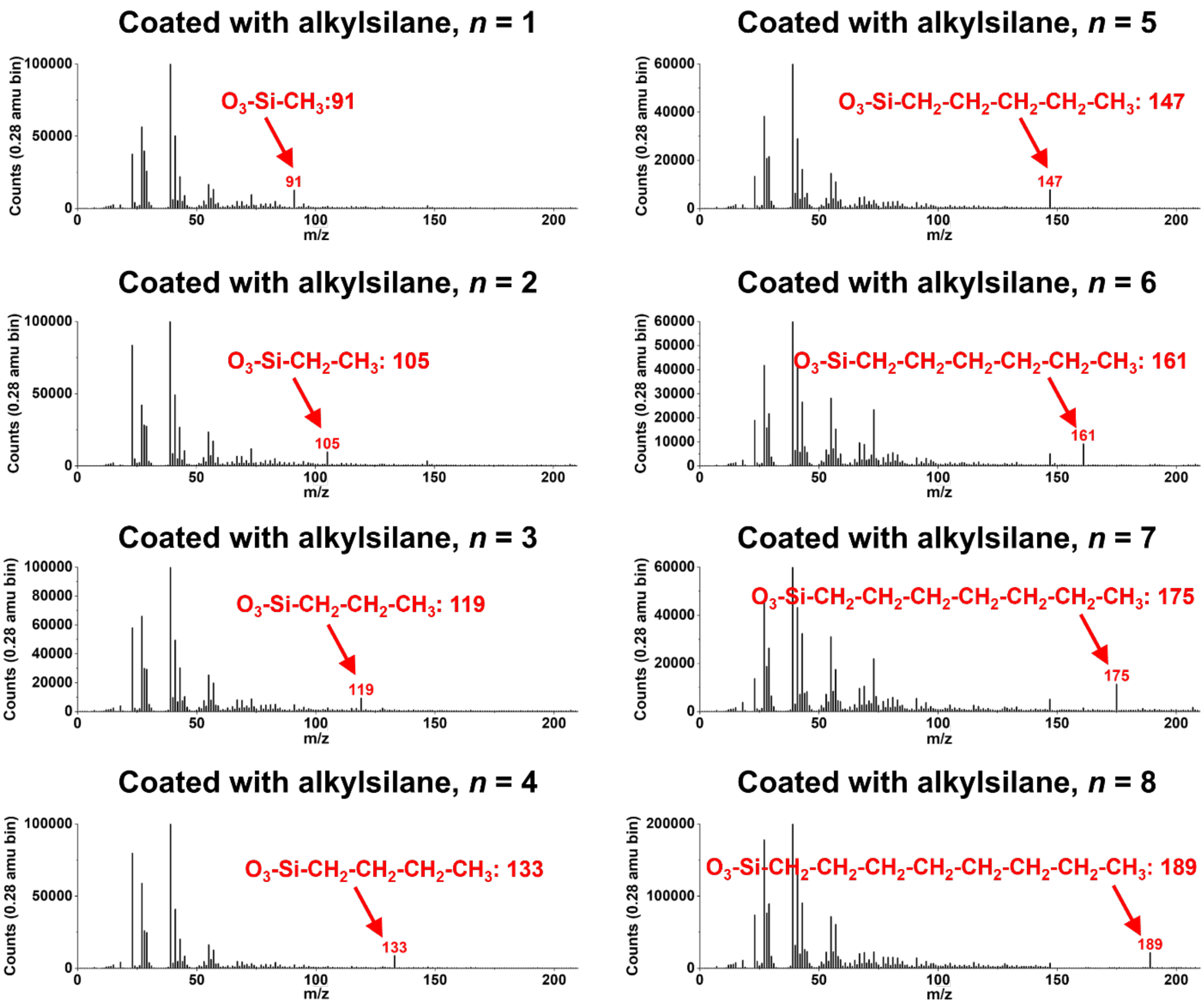

Supplementary Figure 1. Time-of-Flight Secondary Ion Mass Spectrometry (ToF-SIMS) spectra of the surfaces of mica coated with alkylsilanes of *n* = 1 to *n* = 8. Peaks pointed out by the red arrows correspond to the fragments of the alkylsilane, $-O_3-Si-(CH_2)_{n-1}-CH_3$. These peaks showed that the surfaces of mica were successfully coated with the alkylsilanes of the specific *n*. Source data are provided as a Source Data file.

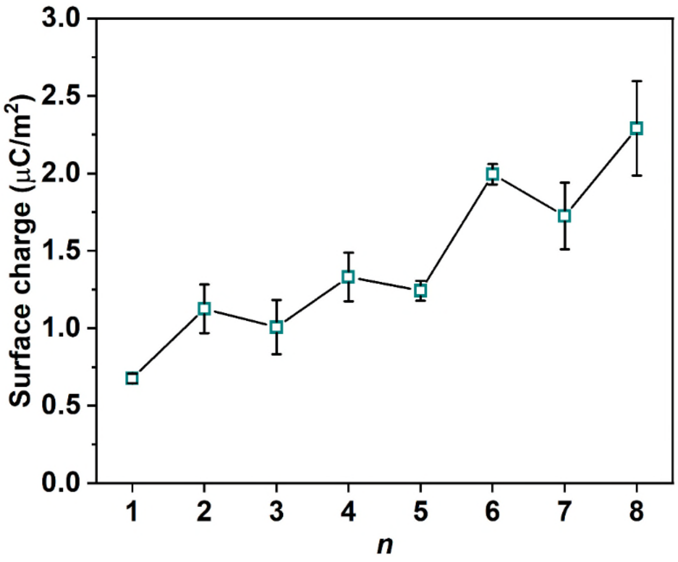

Supplementary Figure 2. Anomalous charging behavior of the alkylsilane-coated surface of mica. When the charges of the alkylsilane-coated surfaces of all $n = 1$ to n = 8 were measured immediately after contact electrification with the uncoated surfaces (i.e., < 2 s), the charges were negative (see Figure 2a of the main text). After leaving the alkylsilane-coated surfaces undisturbed for 10 s under normal ambient conditions after contact electrification, the charges of the alkylsilane-coated surfaces were measured again and found to be positive as shown in this plot. Hence, the polarity of the alkylsilane-coated surface of mica changed from negative to positive after leaving it undisturbed for some time. This anomalous charging behavior has been reported in our previous study[1]. Data are from samples performed in triplicates and are plotted as mean ± standard deviation (SD). Source data are provided as a Source Data file.

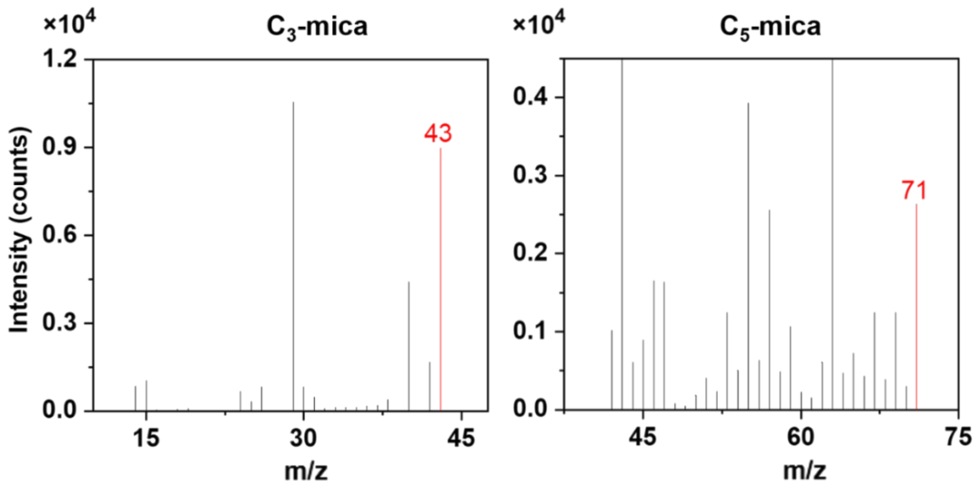

Supplementary Figure 3. Detecting alkyl carbocations. ToF-SIMS spectra that show the analyses of the uncoated pieces of mica after contact electrification with the surfaces that were coated with the alkylsilanes of a specific *n*. The intensities at the m/z values that correspond to the alkyl carbocations, $C_nH_{2n+1}^+$, are labeled in red. Representative surfaces of $n = 3$ (C_3-mica) and $n = 5$ (C_5-mica) are shown. These plots show that the intensities at the m/z values that correspond to the alkyl carbocations are mostly higher than the other intensities; however, they are close to the background signal of typical intensities generated by ToF-SIMS. The strong peaks that correspond to the elements of mica (^{23}Na, ^{27}Al, ^{28}Si, ^{39}K, ^{41}K) have been removed. Source data are provided as a Source Data file.

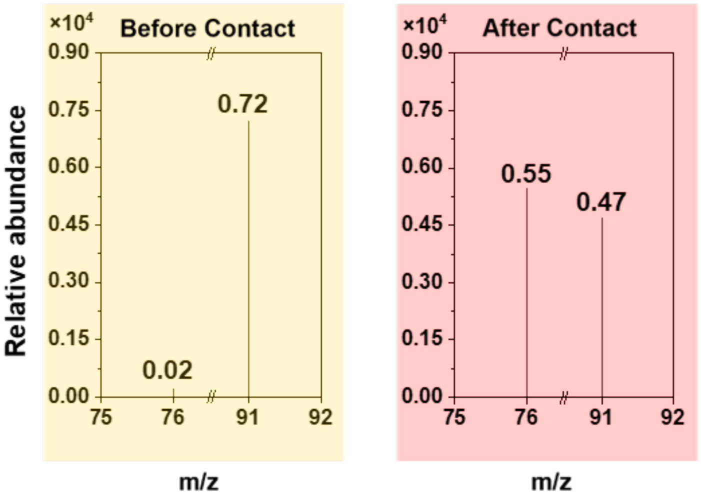

Supplementary Figure 4. ToF-SIMS spectra of the surfaces coated with alkylsilanes of *n* = 1 before (yellow background) and after (pink background) contact electrification with an uncoated surface. m/z = 76 and m/z = 91 correspond to -O_3-Si and -O_3-Si-CH_3 respectively. Before contact electrification, the strong peak at m/z = 91 showed that the alkylsilane, -O_3-Si-CH_3, was coated on the surface. On the other hand, negligible intensity was observed at m/z = 76; hence, the moiety -O_3-Si was not present on the surface. After contact electrification, the peak at m/z = 76 appeared with a strong intensity. On the other hand, the intensity of the peak at m/z = 91 decreased. Hence, the moiety -O_3-Si appeared on the alkylsilanes-coated surface, whereas the amount of -O_3-Si-CH_3 decreased, after contact electrification. These results indicated that contact electrification caused the bond cleavage of Si-C of the alkylsilanes on the surface. Source data are provided as a Source Data file.

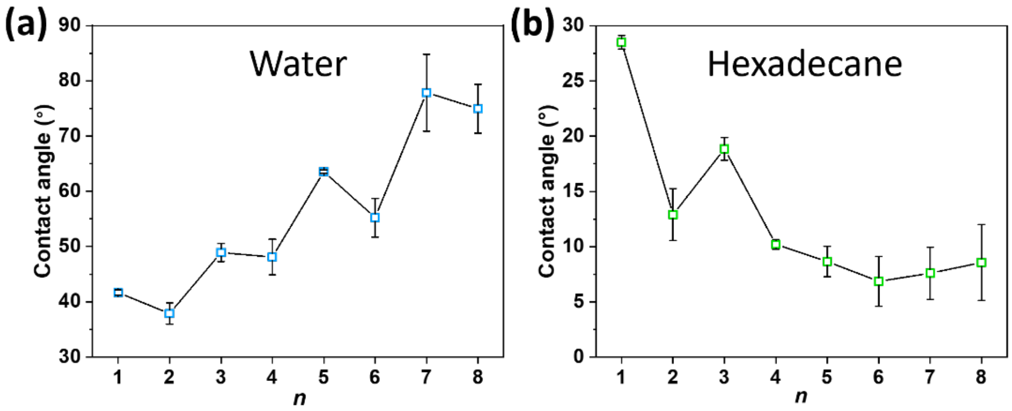

Supplementary Figure 5. Contact angles of (a) water and (b) hexadecane on the surfaces coated with alkylsilanes of different *n*. Previous studies have investigated the contact angles of different types of liquids, including *n*-hexadecane and water, placed on surfaces functionalized with self-assembled monolayers (SAMs) of alkylsilanes[2-5]. These previous studies found that the contact angles are lower when the CH_3-CH_2- moiety at the topmost level of the functionalized molecules on the surface is tilted away from the normal than when it is directed more toward the normal of the surface. Our results in these plots showed that the contact angles of water and hexadecane for $n < 5$ are generally lower for n_{even} than for n_{odd}. Therefore, these results suggested that the CH_3-CH_2- moiety at the topmost surface of the alkylsilanes coated on our surfaces is more tilted away from the normal for n_{even} and more directed toward the normal for n_{odd} (i.e., as illustrated in Figure 1a-c in the main text). Data are from samples performed in triplicates and are plotted as mean ± SD. Source data are provided as a Source Data file.

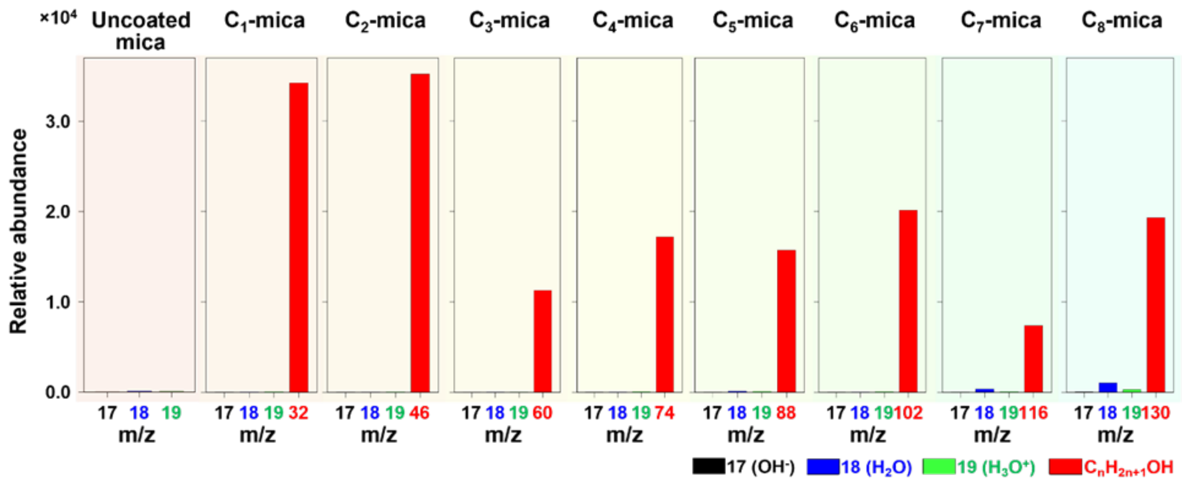

Supplementary Figure 6. Detecting negligible amounts of water on surfaces of mica. ToF-SIMS spectra of the surfaces of uncoated mica before and after contact with C_n-mica for *n* = 1 to *n* = 8. The plots show the peaks corresponding to m/z = 17 (OH^-), 18 (H_2O), 19 (H_3O^+) and $C_nH_{2n+1}OH$. Source data are provided as a Source Data file.

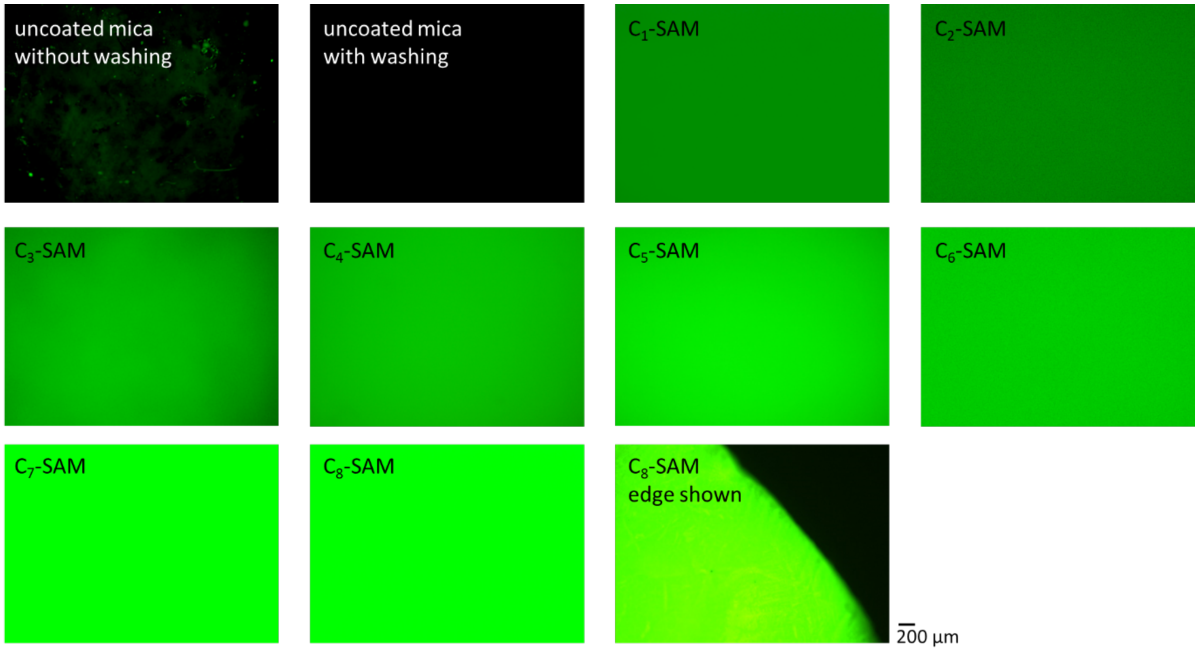

Supplementary Figure 7. Self-assembled monolayers (SAMs) uniformly coated onto surfaces of mica. Fluorescence images of the uncoated mica without washing, uncoated mica with washing, and coated mica, including C_1-SAM, C_2-SAM, C_3-SAM, C_4-SAM, C_5-SAM, C_6-SAM, C_7-SAM, C_8-SAM, C_8-SAM with the edge shown as a contrast.

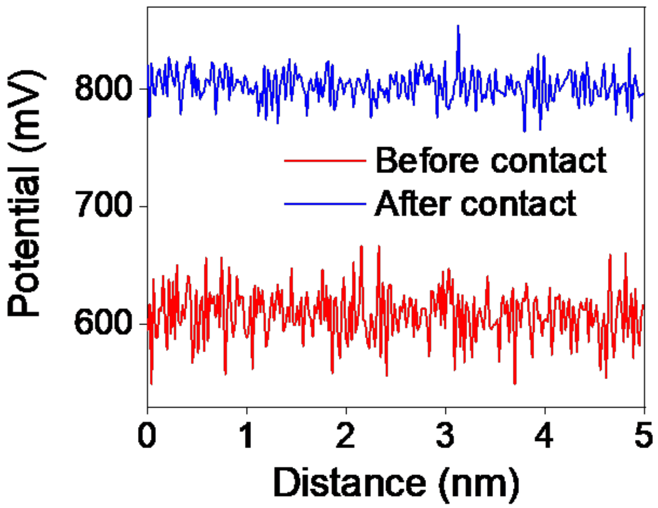

Supplementary Figure 8. Kelvin probe force microscopy (KPFM) analysis of the uncoated mica before and after contact with C_2-SAM. The results showed that the uncoated mica became more positively charged after contact. This result agrees with the measurements of charge using the Faraday cup. Source data are provided as a Source Data file.

References

1 Fang, Y., Chen, L., Sun, Y., Yong, W. P. & Soh, S. Anomalous charging behavior of inorganic materials. *J. Phys. Chem. C* **122**, 11414-11421 (2018).

2 Tao, Y. T. Structural comparison of self-assembled monolayers of n-alkanoic acids on the surfaces of silver, copper, and aluminum. *J. Am. Chem. Soc.* **115**, 4350-4358 (1993).

3 Chang, S.-C., Chao, I. & Tao, Y.-T. Structure of self-assembled monolayers of aromatic-derivatized thiols on evaporated gold and silver surfaces: Implication on packing mechanism. *J. Am. Chem. Soc.* **116**, 6792-6805 (1994).

4 Graupe, M., Takenaga, M., Koini, T., Colorado, R. & Lee, T. R. Oriented surface dipoles strongly influence interfacial wettabilities. *J. Am. Chem. Soc.* **121**, 3222-3223 (1999).

5 Shon, Y.-S., Lee, S., Colorado, R., Perry, S. S. & Lee, T. R. Spiroalkanedithiol-based SAMs reveal unique insight into the wettabilities and frictional properties of organic thin films. *J. Am. Chem. Soc.* **122**, 7556-7563 (2000).
